# Low Heart Rate Is Associated with Cerebral Pulsatility after TIA or Minor Stroke

**DOI:** 10.1002/ana.26480

**Published:** 2022-08-29

**Authors:** Alastair J.S. Webb, Karolina A. Wartolowska, Linxin Li, Peter M. Rothwell

**Affiliations:** ^1^ Wolfson Centre for Prevention of Stroke and Dementia University of Oxford Oxford UK

## Abstract

**Objective:**

Beta‐blockers are beneficial in coronary artery disease but less so in stroke prevention and dementia, potentially due to reduced heart rate (HR). Cerebral pulsatility is strongly associated with cerebral small vessel disease (SVD) and may be increased by lower diastolic pressures resulting from longer cardiac cycles.

**Methods:**

Patients 4–6 weeks after TIA or non‐disabling stroke (Oxford Vascular Study) underwent 5 minutes continuous monitoring of blood pressure (BP), electrocardiogram (ECG), and middle cerebral artery flow velocity (transcranial ultrasound). Beat‐to‐beat relationships between HR, blood pressure and Gosling's pulsatility index (MCA‐PI) are reported as beta‐coefficients from general linear models for each individual.

**Results:**

Across 759 patients, average MCA‐PI during monitoring was associated with lower HR and diastolic BP (DBP) and greater systolic BP (SBP) (∆MCA‐PI per 10 bpm/mmHg: −0.02, −0.04, 0.03, all *p* < 0.001), with HR particularly associated with low end‐diastolic cerebral velocity (0.86, *p* = 0.014). Beat‐to‐beat HR was strongly associated with concurrent low DBP and high SBP, potentially mediating the association with greater beat‐to‐beat cerebral pulsatility (average ∆MCA‐PI vs HR/DBP/SBP unadjusted: −0.062, −0.052, 0.0092; adjusted for concurrent BP: −0.039, −0.11, 0.041). The beat‐to‐beat association between HR and MCA‐PI increased with age, beta‐blockers, arterial stiffness, low HR (age > 70 + HR < 65 vs age < 70 + HR > 65: −0.081 vs −0.024, interaction *p* < 0.001), and severe SVD on MRI (age > 70 + severe vs age < 70 + none: −0.087 vs −0.047, interaction *p* = 0.03), with interactions between age, severe SVD, and low HR synergistically increasing MCA‐PI.

**Interpretation:**

Low HR is associated with greater cerebral pulsatility in patients with SVD, potentially mediated by lower diastolic blood flow and representing a novel potential treatment target. ANN NEUROL 2022;92:909–920

## Introduction

Elevated heart rate (HR) is associated with an increased risk of all‐cause mortality and cardiovascular events^1^ reflecting its association with poor cardiovascular fitness,^2^ sympathetic overdrive,^3^ obesity, and hypertension,^4^ whereas its reduction with beta‐blockers after myocardial infarction^5^ or heart failure^6^ is associated with improved cardiac outcomes. However, these studies have largely been performed in younger populations with limited cerebrovascular disease. Beta‐blockers are less effective in primary prevention of stroke than non‐rate‐limiting antihypertensives, despite similar reductions in blood pressure,^7^ such that they are fifth‐line options for hypertension. Furthermore, use of beta‐blockers has been associated with an increase in the risk of vascular dementia,^8^, ^9^ and a higher risk of progression to functional dependence in patients with cognitive impairment.^10^

Cerebral arterial pulsatility is strongly associated with severity of cerebral small vessel disease (SVD),^11^ manifest most often as white matter hyperintensities (WMH) on MRI,^12^ which are associated with 30% of strokes and 45% of dementia.^13^ Cerebral pulsatility reflects enhanced transmission of the pulsatile aortic waveform to the brain through increasingly stiff vessels.^14^ Following the transition to the late‐life hypertensive phenotype of increased aortic stiffness above 55 years of age,^15^ it progresses in parallel with aortic pulsatility, aortic stiffness,^16^ and SVD,^17^, ^18^, ^19^ and may play a key role in pathogenesis of SVD and its consequences, through either excessive shear stress during systole or hypoperfusion during diastole.

A low HR may increase cerebral arterial pulsatility by causing a lower diastolic pressure, especially in the cerebral circulation. During a cardiac cycle, once the pressure wave of systolic contraction has passed, the blood pressure falls until the next cardiac contraction. As such, DBP falls further with lower HRs and longer cardiac cycles. In normal vessels, the diastolic pressure is sustained by elastic recoil of the aorta returning some of the energy of systolic contraction to the blood (“Windkessel” effect) and by reflection of the outgoing systolic pressure wave from points of distal resistance producing a reverse travelling wave that reaches the aorta in diastole, enhancing the diastolic pressure. However, in stiffer, older vessels, the effect of long cardiac cycles on DBP may be exacerbated by reduced elastic recoil and the reverse wave arriving earlier in systole, resulting in less support for the diastolic pressure.^11^ This low diastolic pressure is then further reduced during transmission to the brain through stiff vessels, where increasing distal resistance at older ages may have a synergistic effect on late diastole, further reducing end‐diastolic cerebral blood flow.

Therefore, we hypothesized that, although a lower HR may be beneficial for prevention of cardiac events, it may be associated with increased cerebral pulsatility in elderly patients with stiff vessels. In the OXVASC^20^ population‐based study, we determined the beat‐to‐beat effect of HR fluctuations on concurrent blood pressure and cerebral pulsatility, stratified by severity of cerebral SVD.

## Methods

### Study Population

Consecutive, consenting patients with transient ischemic attack (TIA) or minor stroke were recruited between September 2010 and September 2019, as part of the Phenotyped Cohort of the Oxford Vascular Study (OXVASC).^20^ All patients within a defined population in Oxfordshire with acute cerebrovascular events are identified, and all those with TIA or minor events not requiring admission to hospital are reviewed at the OXVASC daily emergency assessment clinic, following a referral after attendance at the emergency department or from primary care, usually within 24 hours. The OXVASC population consists of >92,000 individuals registered with about 100 primary‐care physicians in Oxfordshire, United Kingdom. All consenting patients underwent a standardised medical history and examination, ECG, blood tests and a stroke protocol MRI brain and contrast‐enhanced MRA (or CT‐brain and carotid Doppler ultrasound or CT‐angiogram), an echocardiogram and 5 day ambulatory cardiac monitor. Patients were assessed by a study physician, reviewed by the senior study neurologist (P.M.R.) and followed‐up face‐to‐face at 1, 3, 6 and 12 months, and 2, 5 and 10 years. Medication is prescribed according to guidelines, most commonly with dual antiplatelets (aspirin and clopidogrel) for 1 month, high dose statins (atorvastatin 40–80 mg) and a combination of perindopril and indapamide. Participants are excluded from certain physiological tests if they are under 18 years, too cognitively impaired to consent, pregnant, have a recent myocardial infarction, unstable angina, heart failure (New York Heart Association [NYHA] 3–4 or ejection fraction <40%) or untreated bilateral carotid stenosis (>70%). OXVASC is approved by the Oxfordshire Research Ethics Committee.

### Physiological Assessment

In a quiet, dimly‐lit, temperature‐controlled room (21‐23°C), after approximately 15 minutes supine rest, patients underwent physiological monitoring over 5 minutes at the ascertainment visit or 1 month clinic, consistent with recommendations for monitoring effects of variability in HR,^21^ and previous analyses in this population of the validity and reproducibility of blood pressure^22^ and cerebral blood flow monitoring over 5 minutes.^23^ Continuous 3‐lead electrocardiogram (ECG) and non‐invasive finger arterial blood pressure (ABP) were acquired (Finometer MIDI, Finapres Medical Systems) via a Powerlab 8/35 (ADInstruments). Automated calibration (Physiocal) was performed until the recording was stable, but turned off during each test. Estimated brachial waveforms were calibrated offline by linear regression to 2–3 supine, oscillometric brachial readings, performed immediately prior to the monitoring period on the contralateral arm, with manual exclusion of artifacts. Waveforms were preferentially recorded from the middle phalanx of the middle finger although the finger cuff could be moved to an adjacent finger or the proximal phalanx of the same finger as required and the hand could be warmed.

Cerebral blood flow velocity (CBFV) from bilateral middle cerebral arteries (MCAs) was simultaneously measured using transcranial Doppler (TCD) ultrasound (DWL Dopplerbox; Compumedics DWL, Singen, Germany) with a 2‐MHz probe at the temporal bone window held by a Diamon monitoring headset, recording the highest velocity trace between 50 and 55 mm depth. BP and ECG waveforms were acquired at 200 Hz and TCD at 100 Hz. Consecutive beat‐to‐beat signals of BP and CBFV from bilateral MCAs were median filtered (7 data points), with automatic detection and linear interpolation of ectopic beats, and were then visually reviewed by an experienced operator (A.J.S.W.) for blinded quality assessment with linear interpolation of artifacts.

Applanation tonometry (Sphygmocor, AtCor Medical, Sydney, Australia) was used to measure carotid‐femoral pulse wave velocity (aortic‐PWV) and aortic systolic and diastolic BP and pulse pressure (ao‐SBP, ao‐DBP, ao‐PP).

### Statistical Analysis

Demographic characteristics of the population were described as mean and standard deviation, or as median and interquartile range for non‐normal characteristics. Cross‐sectional associations were derived between average HR, BP, and CBFV during monitoring by general linear models, unadjusted; adjusted for age, gender, and cardiovascular risk factors (diabetes, history of hypertension, smoking, and dyslipidemia) and adjusted for blood pressure. Associations were expressed per 10 unit change in the predictor (HR, DBP, or SBP). Each predictor was also stratified into quintiles, and the mean value and confidence interval within each quintile calculated.

For each patient, beat‐to‐beat associations between change in HR, SBP, or DBP with cerebral indices (peak systolic velocity [PSV], end‐diastolic velocity [EDV], MCA‐PI) were determined by general linear models, with and without adjustment for the other concurrent predictors (HR, SBP, or DBP). Distributions of these beta‐coefficients were plotted as kernel density plots, and were averaged across the entire population, across those in whom the beat‐to‐beat association was significant (*p* < 0.05), by quintile of systemic characteristics (age, PWV, SBP, DBP, PP) and for participants using each specific class of antihypertensive medication compared to patients not using that class of medication. Factors predicting the presence of a significant association (*p* < 0.05) were identified by logistic regression.

For each patient, the degree of mediation of the beat‐to‐beat relationship between HR, SBP, or DBP with each cerebral blood flow index by each of HR, SBP, and DBP was determined by mediation analysis. Mediation assumes a temporal relationship between variables to support putative causation. This is provided in this analysis by the physiological pathway the blood follows from the heart, to the aorta (blood pressure) to the brain, with later measures physiologically dependent upon earlier variables. Each relationship was classified as no association, a purely direct association, an association that was fully mediated by another index or an association that was partially direct and partially mediated.

Severity of WMH on MRI were determined by an experienced reviewer (L.L.) according to the Fazekas score and classified as none, mild,^1^, ^2^ moderate^3^, ^4^ or severe,^5^, ^6^ blind to physiological results. Further analyses were performed stratified by severity of cerebral microbleeds, presence of lacunes, deep perivascular spaces, and total SVD score, as previously reported.^24^ Mean HR, MCA‐PI, and beta‐coefficients for beat‐to‐beat associations were stratified by severity of WMH. The reliability, reproducibility, and clinical significance of these measures have previously been reported in this population,^25^ with previous reports detailing the burden of all forms of SVD in this population.^26^ Average associations between beta‐coefficients for within‐individual associations between HR and PI were determined by severity of SVD, age, gender, cardiovascular risk factors, and baseline HR, with and without adjustment for cardiovascular risk factors, and with and without interactions between severity of WMH, age, and baseline HR.

Analyses were performed in R and Matlab r2018, using in‐house software.

## Results

### Patient Population

The study included 759 patients with good or adequate quality BP and transcranial ultrasound monitoring, allowing calculation of the beat‐to‐beat relationship between HR and pulsatility index (PI), with 808 patients with measures of aortic stiffness. A total of 497 patients (65%) had a stroke, whereas 262 had a TIA (35%). Demographics of the population are shown in Table 1.

**TABLE 1:** Demographic Characteristics of Patients included in the Study

	PP	PI
N	805	759
Age	66.1 (13.5)	65.8 (13.5)
Female	310 (38.5)	287 (37.8)
Hypertension	560 (69.6)	527 (69.4)
Diabetes	78 (9.7)	76 (10)
Atrial fibrillation	47 (5.8)	43 (5.7)
Dyslipidemia	239 (29.7)	221 (29.1)
Family history stroke	207 (25.7)	196 (25.8)
Ever smoker	390 (48.4)	370 (48.7)
Weight (Kg)	79.9 (33.9)	80.1 (34.7)
BMI (kg/m^2^)	27 (5.2)	27 (5.3)
Cholesterol	5.1 (1.3)	5.1 (1.3)
Creatinine	77.7 (21.4)	77.7 (21.2)
PWV (m/s)	9.7 (2.9)	9.6 (2.8)
Aortic SBP (mmHg)	120.7 (17.7)	120.6 (17.6)
Aortic DBP (mmHg)	73 (9.4)	73.1 (9.4)
Brachial SBP (mmHg)	124.4 (18.1)	124.4 (18.1)
Brachial DBP (mmHg)	68.3 (10.5)	68.3 (10.5)
HR (bpm)	65.7 (10.8)	65.7 (10.8)

### Between‐Individual Associations between HR or BP with Cerebral Pulsatility

In cross‐sectional analyses, a higher average MCA‐PI during monitoring was associated with a lower average HR, higher SBP, and lower DBP, although the magnitude of the effect was limited (Table 2). A 10 mmHg increase in SBP was associated with a 0.03 increase in PI (0.18% of a standard deviation), a 10 mmHg rise in DBP was associated with a 0.04 lower PI (−0.23% of a standard deviation) and a 10 bpm increase in HR was associated with a 0.02 lower PI (−0.12% of a standard deviation). For cerebral blood flow indices, cerebral pulsatility was predominantly related to a lower EDV, to a greater extent than the positive association with PSV. Results were similar after adjustment for risk factors and other BP indices (Table 2).

**TABLE 2:** Cross‐Sectional Associations between Systemic BP and HR with Cerebral Blood Flow

	PI b	PI p	PSV b	PSV p	EDV b	EDV p	MV b	MV p
Univariate								
HR	−0.02 (−0.034–−0.011)	0.001	0.08 (−1.1–1.3)	0.99	0.86 (0.24–1.5)	0.014	0.58 (−0.19–1.4)	0.21
SBP	0.03 (0.023–0.037)	<0.001	0.38 (−0.34–1.1)	0.32	−0.87 (−1.2–−0.5)	<0.001	−0.44 (−0.9–0.021)	0.061
DBP	−0.04 (−0.055–−0.032)	<0.001	−2 (−3.2–−0.74)	0.002	0.36 (−0.27–0.99)	0.32	−0.4 (−1.2–0.4)	0.314
PWV	0.32 (0.28–0.36)	<0.001	−2.1 (−6.9–2.7)	0.25	−12 (−14–−9.3)	<0.001	−8.4 (−11–−5.4)	<0.001
Adjusted for Age, Gender and Cardiovascular Risk Factors								
HR	−0.03 (−0.038–−0.018)	<0.001	−0.66 (−1.9–0.62)	0.306	0.68 (0.11–1.2)	0.149	0.21 (−0.56–0.99)	0.911
SBP	0.02 (0.011–0.023)	<0.001	0.64 (−0.15–1.4)	0.133	−0.3 (−0.65–0.05)	0.075	0.03 (−0.45–0.51)	0.993
DBP	−0.03 (−0.041–−0.021)	<0.001	−1.9 (−3.2–−0.55)	0.007	−0.01 (−0.61–0.58)	0.949	−0.61 (−1.4–0.2)	0.169
PWV	0.15 (0.094–0.2)	<0.001	2.8 (−3.8–9.4)	0.428	−3 (−5.9–−0.09)	0.013	−1.1 (−5.1–2.8)	0.413
Adjusted for Age, Gender and Concurrent SBP and DBP								
HR	−0.02 (−0.026–−0.009)	<0.001	−0.49 (−1.7–0.74)	0.186	0.42 (−0.14–0.99)	0.054	0.1 (−0.67–0.86)	0.857
SBP	0.05 (0.046–0.06)	<0.001	2.4 (1.4–3.4)	0.271	−0.6 (−1.1–−0.13)	0.09	0.4 (−0.23–1)	0.863
DBP	−0.09 (−0.10–−0.079)	<0.001	−4.4 (−6.2–−2.7)	0.002	0.72 (−0.079–1.5)	0.654	−0.98 (−2.1–0.09)	0.068
PWV	0.09 (0.046–0.14)	<0.001	−0.08 (−6.7–6.5)	0.428	−2.5 (−5.5–0.5)	0.013	−1.8 (−5.9–2.3)	0.413

Each model presents the beta‐coefficient and p‐value for the general linear model between each predictor and each outcome, without (univariate) adjustment, adjusted for age, gender, diabetes, smoking, dyslipidemia, and hypertension or adjusted for age, gender, SBP and DBP.

b = unstandardized beta‐coefficient for change in the cerebral index per 10 unit change in the systemic index.

Mean PSV was weakly associated with blood pressure indices, with an association only between a higher DBP and higher PSV. In contrast, EDV was associated with HR, arterial stiffness and SBP, although this diminished after adjustment (Table 3).

**TABLE 3:** Beat‐to‐beat relationships between systemic BP and HR with cerebral blood flow

	PI b	PI sig	PI beta (sig)	PSV b	PSV sig	PSV beta (sig)	EDV b	EDV sig	EDV beta (sig)
Univariate									
HR	−0.062 (−0.07–−0.06)	83	−0.072 (−0.08–−0.07)	1.9 (1.5–2.3)	66	2.8 (2.2–3.4)	2.8 (2.6–3.1)	84	3.3 (3–3.6)
SBP	−0.0092 (−0.01–−0.007)	63	−0.014 (−0.017–−0.01)	1.8 (1.6–2.)	80	2.2 (2.0–2.4)	1.2 (1.1–1.3)	83	1.4 (1.3–1.5)
DBP	−0.052 (−0.056–−0.05)	78	−0.065 (−0.07–−0.06)	2.6 (2.3–2.9)	74	3.5 (3.1–3.8)	2.9 (2.7–3.1)	89	3.3 (3.1–3.5)
Multivariate									
HR	−0.039 (−0.043–−0.036)	65	−0.057 (−0.06–−0.05)	1.5 (1.1–1.9)	62	2.4 (1.8–3)	1.9 (1.7–2.2)	75	2.6 (2.3–2.9)
SBP	0.041 (0.038–0.044)	73	0.054 (0.05–0.057)	2.1 (1.8–2.4)	75	2.7 (2.4–3.1)	−0.35 (−0.5–−0.18)	66	−0.52 (−0.8–−0.26)
DBP	−0.11 (−0.11–−0.10)	80	−0.13 (−0.13–−0.12)	−0.93 (−1.5–−0.4)	66	−1.3 (−2–−0.47)	3 (2.7–3.4)	75	4 (3.5–4.4)

Mean, beta‐coefficients for the beat‐to‐beat relationship between HR, SBP, and DBP with beat‐to‐beat change in middle cerebral artery PI, PSV, mean (MV) and trough (EDV) blood flow velocities. Values reflect change in the cerebral value for each 10 unit change in HR or BP, with or without beat‐to‐beat adjustment for concurrent SBP and DBP.

### Beat‐to‐Beat Associations between HR and BP with Cerebral Pulsatility

Within‐individual, beat‐to‐beat associations between HR and cerebral PI were stronger than cross‐sectional associations between average values during monitoring. There was a 0.062 fall in PI for a 10 bpm increase in HR (Table 3), equating to nearly an entire standard deviation fall in PI reduction per 10 bpm rise in HR (97.5% of a standard deviation), although there was a moderate reduction in the magnitude of the relationship after adjustment for concurrent BP changes (Table 3). There was a negative skew to the distribution of the relationships between DBP or HR and MCA‐PI.

**FIGURE 1:**
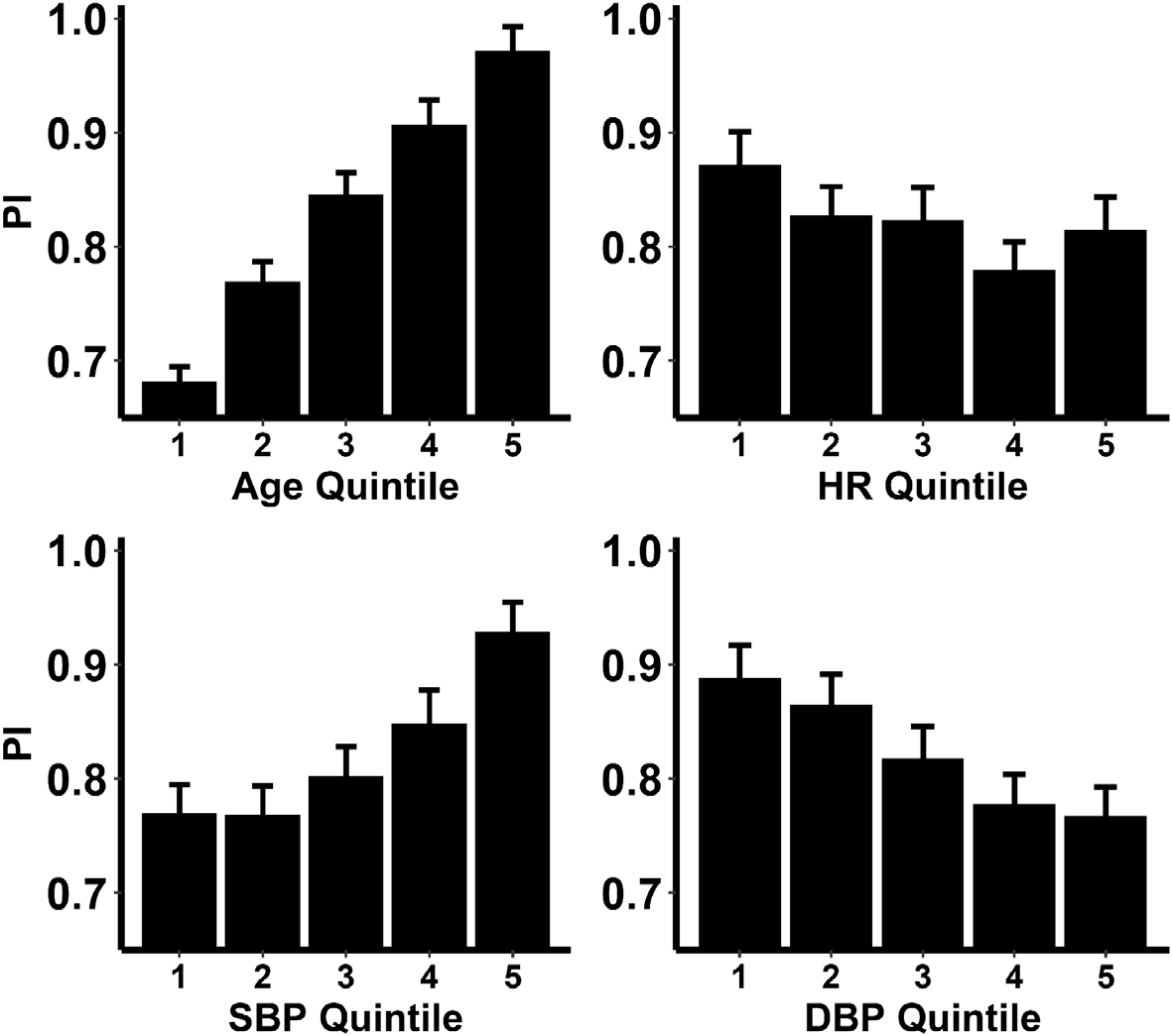
Cross‐sectional relationships between mean demographic indices and mean cerebral pulsatility index (PI) during monitoring, per 10 mmHg increase in BP or 10 bpm increase in heart rate (HR). Columns represent mean value for each quintile, with 95% confidence intervals.

Increases in beat‐to‐beat HR, SBP, or DBP were all associated with an increase in PSV or EDV, with a stronger relationship between HR and EDV than HR and PSV, although relationships between HR and cerebral blood flow indices diminished upon adjustment for blood pressure changes. However, there was a stronger relationship between BP level and CBFV than with HR, in contrast to a stronger relationship between HR and MCA‐PI (Fig 1).

### Determinants of Relationships between HR or BP with Cerebral Pulsatility

There was significant heterogeneity between patients (p < 0.001) and not all patients had a significant beat‐to‐beat relationship between HR and PI, although relationships were similar in men and women. When limiting the analysis to patients with a significant beat‐to‐beat association, the magnitude of the association between HR and pulsatility rose to 0.072 per 10 bpm (unadjusted) and 0.057 following adjustment for beat‐to‐beat blood pressure changes (Table 3).

A similar proportion of patients had significant beat‐to‐beat relationships between SBP and PSV, or DBP and EDV (Table 3), with a small increase in the magnitude of this relationship when restricted to those patients with significant relationships. Associations between SBP and PSV were largely consistent across quintiles of age, HR, or other physiological measures but the relationship between DBP and EDV was stronger in older patients with increased arterial stiffness (Fig 3). In contrast the within‐individual, beat‐to‐beat relationships between SBP and DBP with PI were stronger in younger patients.

The strength and magnitude of the relationship between HR and PI increased with increasing arterial stiffness. There was a strong cross‐sectional relationship between MCA‐PI and either PWV (beta = 0.03, *p* < 0.001) or aortic PP (beta = 0.006, p < 0.001) across the population, whereas increases in either PWV or aortic PP were associated with a greater beat‐to‐beat relationship between HR and MCA‐PI (Fig 3), particularly for aortic PP.

**FIGURE 2:**
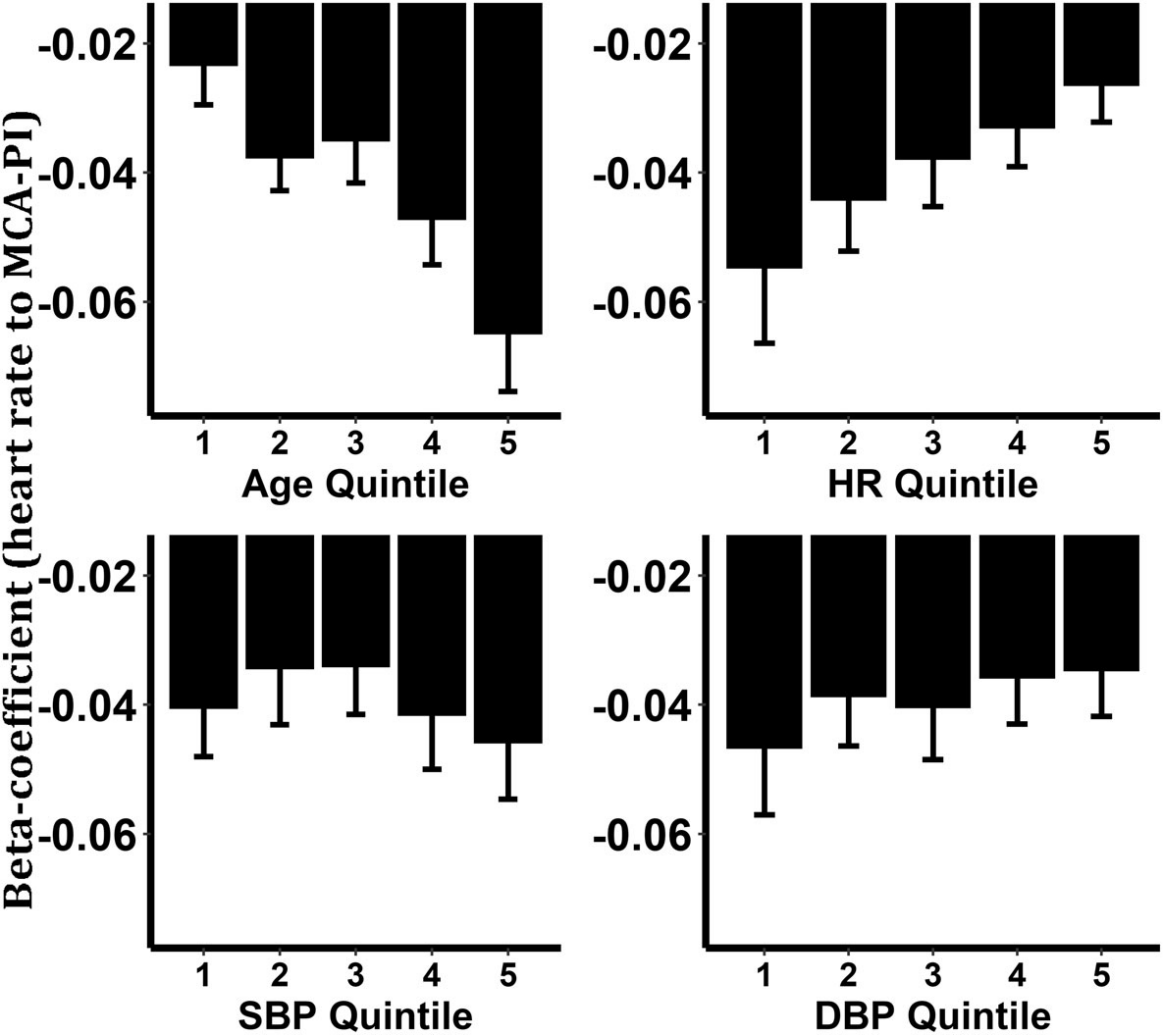
Beat‐to‐beat relationship between HR and cerebral PI, stratified by demographic indices, per 10 mmHg increase in BP or 10 bpm increase in HR. Columns represent mean value for each quintile, with 95% confidence intervals.

The magnitude of the beat‐to‐beat relationship between HR and MCA‐PI was also greater in older patients and patients with a lower HR (Fig 2), for both men and women. As such, patients above 70 years and below the median HR (65) had a significantly stronger relationship between HR and PI in adjusted (−0.081) and unadjusted (−0.059) associations, compared to younger patients with higher HRs (unadjusted = −0.039; adjusted = −0.024).

**FIGURE 3:**
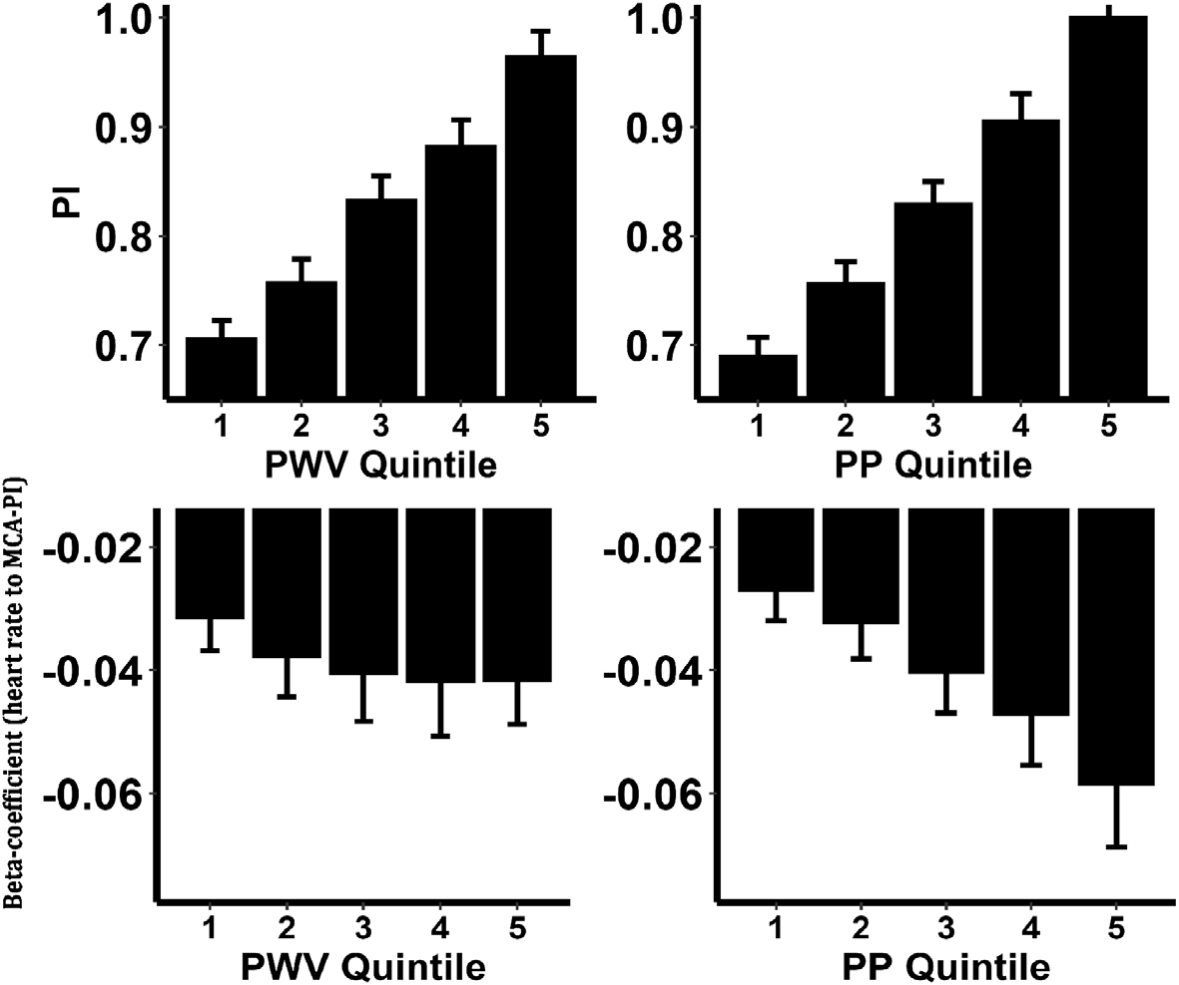
Relationships between HR and cerebral PI, stratified by markers of arterial stiffness. Panels (A) and (B) show the average middle cerebral pulsatility (MCA‐PI) across the population, stratified by quintile of aortic pulse wave velelocity (PWV) or aortic pulse pressure (PP). C,D, The beat‐to‐beat relationship between a 10 bpm increase in HR and change in MCA‐PI, stratified by quintile of PWV and PP. Errors bars show the 95% confidence intervals.

**FIGURE 4:**
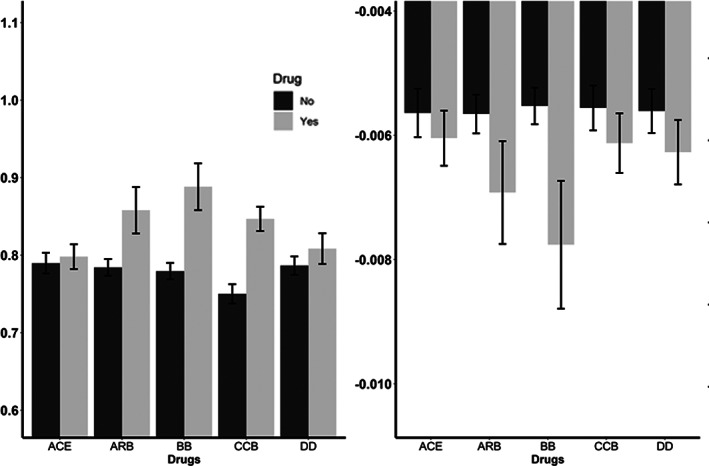
Relationship between use of different classes of antihypertensive medication and the between‐individual difference in mean MCA‐PI during monitoring (A) and the beat‐to‐beat relationship between HR and PI during monitoring, both unadjusted (B) or adjusted for other BP indices (C). ACE = angiotensin converting enzyme inhibitor; ARB = angiotensin receptor blocker; BB = beta‐blocker; CCB = calcium channel 1locker; DD = diuretic.

**FIGURE 5:**
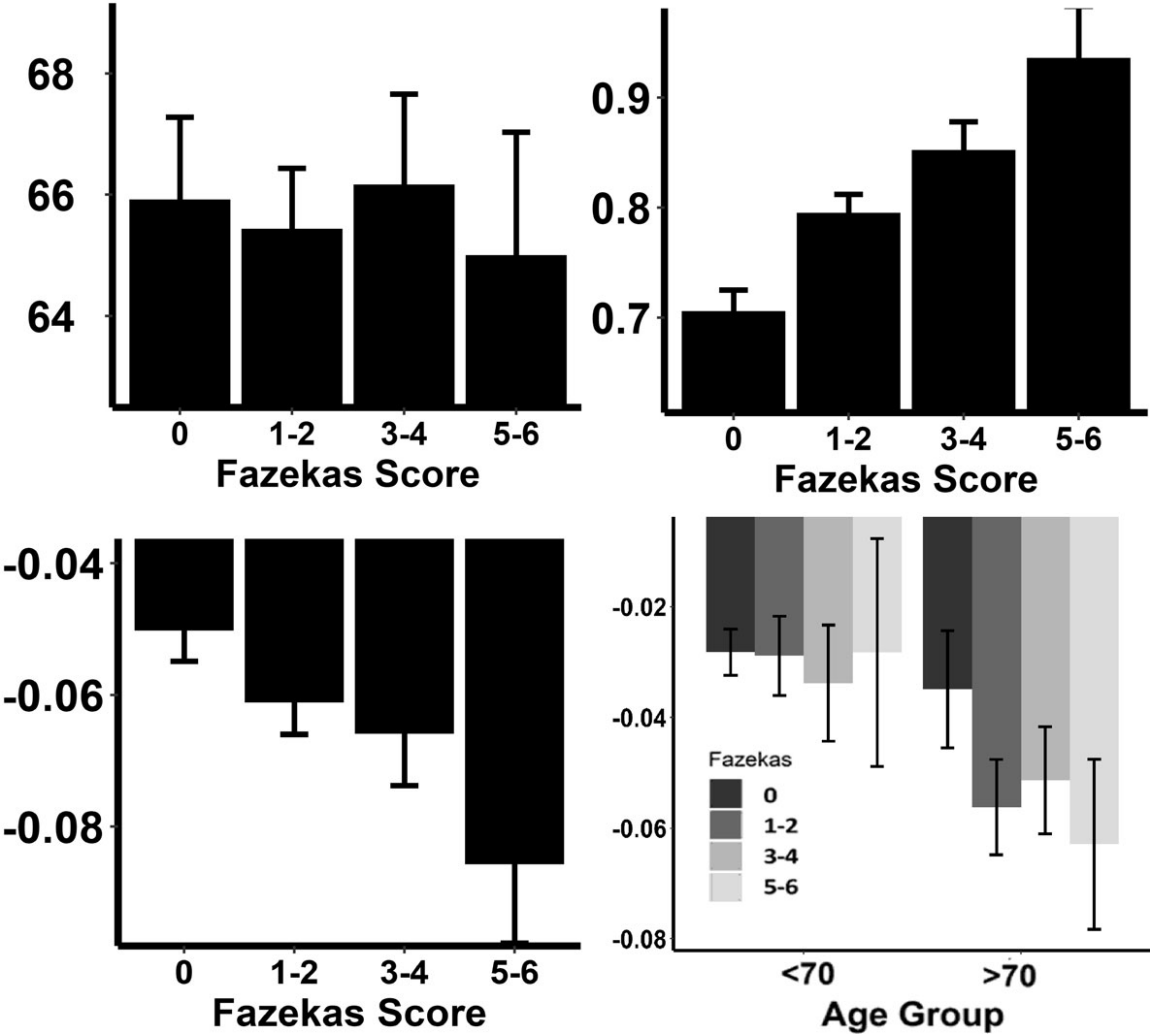
The relationship between HR and cerebral pulsatility, stratified by markers of SVD. Panels (A) and (B) show the average HR and cerebral PI stratified by severity of WMH. Panels (C) and (D) demonstrate the average change in PI per 10 bpm change in HR during beat‐to‐beat monitoring, stratified by the severity of WMH and further stratified by age above or below the median of the population (D).

### Effects of Antihypertensive Medication on the Relationship between HR and MCA‐PI

Current medication use affected the relationship between HR and MCA‐PI, with a significantly stronger relationship in patients on beta‐blockers, calcium channel blockers, or angiotensin‐receptor blocker compared to patients not using those medication classes, with the greatest difference in patients on beta‐blockers (Fig 4). However, the majority of patients were on more than one antihypertensive agent, confounding comparisons between antihypertensive classes, preventing reliable conclusions.

### Mediation of the Relationship between HR and MCA‐PI

HR was associated with pulse pressure, and both were associated with MCA‐PI, both cross‐sectionally between individuals and for individual, beat‐to‐beat changes. However, there was minimal evidence that the relationship between HR and MCA‐PI was directly mediated by pulse pressure with the commonest relationship being a direct association between HR and MCA‐PI, followed by no association, with only weak mediation by PP. In contrast, the relationship between HR and absolute cerebral blood flow velocities was mediated by absolute blood pressure levels, especially for DBP, with the relationship between a low HR and a low EDV being significantly mediated by its effects on low DBP (31%).

### Effect of Severity of Cerebral SVD on the Relationship between HR and MCA‐PI

A total of 662/759 (87%) of patients with valid physiological monitoring had MRI imaging at baseline. Increased Fazekas score was strongly associated with cerebral pulsatility in cross‐sectional analyses (Fig 5, adjusted odds ratio [OR] per SD = 1.26, *p* = 0.021, ordinal regression), although there was no cross‐sectional association with average HR. However, there was a significantly stronger beat‐to‐beat relationship between HR and MCA‐PI in patients with severe WMH compared to no WMH (unadjusted −0.080 vs −0.047, *p* < 0.0001; adjusted for age, gender, and cardiovascular risk factors: −0.073 vs −0.055 *p* = 0.027), with a significant interaction between age and severe WMH (age > 70 + severe versus age < 70 + none: −0.087 vs −0.047, interaction *p* = 0.03). When considering other markers of SVD, the relationship between HR and MCA‐PI also increased with increasing perivascular spaces in the basal ganglia (*p* = 0.003), although this was not significant after adjustment for age and gender. There was no significant increase in the relationship between HR and MCA‐PI with increasing number of microbleeds or the presence of lacunar strokes, although the prevalence of these was less than with WMH.^26^ Overall, there was a significant, synergistic interaction with age (*p* = 0.03), and a borderline 3‐way synergistic interaction between severe WMH, age, and HR (*p* = 0.06) with increased MCA‐PI with increased age and WMH and lower HR.

## Discussion

In a population of patients with a recent TIA or minor stroke, a lower HR was associated with increased cerebral pulsatility. Although there was a relatively weak cross‐sectional association for average values across the population, there was a much stronger relationship for beat‐to‐beat changes between HR and resulting MCA‐PI. Beat‐to‐beat MCA‐PI was more strongly associated with lower end‐diastolic cerebral blood flow than raised systolic flow, whereas effects of HR on EDV were mediated by DBP. The relationship between low HR and increased MCA‐PI was greater in patients who were older, had increased arterial stiffness, were on beta‐blockers or had lower mean HRs, and was particularly strong in patients with severe cerebral SVD, with synergistic interactions between age, low HR and severe cerebral SVD.

Cerebral SVD is the most prevalent cause of significant neurological disability in developed nations. It is associated with 30% of ischemic strokes, 80% of hemorrhagic strokes and 45% of dementia, as well as late‐onset refractory depression^27^ and an increased risk of functional dependence.^28^ It is manifest on brain imaging as increased WMH, lacunar strokes, dilated CSF‐filled perivascular spaces around vessels, and areas of microscopic hemosiderin deposition (microbleeds).^12^ WMH affect over 50% of patients over 65 years and almost all patients over 80,^25^ and are strongly associated with historic midlife hypertension and late life pulsatile blood pressure,^29^ reflecting the transition to an arterial stiffness dependent late‐life hypertensive phenotype,^15^ with a particular effect of pulsatile pressure in the posterior circulation.^19^

The only intervention shown to reduce WMH progression is a moderate effect of blood pressure control to normotensive levels.^30^ However, SVD is also strongly associated with arterial stiffness and cerebral arterial pulsatility,^11^, ^31^ reflecting transmission of pulsatile blood flow from the aorta through stiff vessels to the brain. Pulsatility progresses above 55 years,^14^, ^32^ in parallel with progression of SVD, is highly reproducible over 5 years within‐individuals, and is a biologically plausible mechanism for inducing cSVD, both through increased sheer stress due to excessive systolic pressures and hypoperfusion during diastole. Vasodilating drugs with beneficial effects on stroke^33^ risk also reduced cerebral pulsatility,^34^ such as cilostazol in the ECLIPSE trial,^35^ but vasodilators, and PDE3 inhibitors in particular, also increase HR rather than just lowering BP, suggesting a potential alternative mechanism of action.

Our study supports the hypothesis that increases in HR are associated with reduced cerebral pulsatility, explicable by a shorter period for diastolic blood flow to fall during shorter cardiac cycles. We also demonstrated that a reduction in BP is consistent with a beat‐to‐beat reduction in cerebral pulsatility, as expected if SBP is proportionally reduced more than DBP. If this hypothesis is correct, the association with HR would be proportionally greater at a lower resting HR, as was the case in our population and consistent with a stronger association between HR and PI in patients on beta‐blockers in this study, and the greater pulsatility on beta‐blockers compared to amlodipine in previous studies.^36^, ^37^ Furthermore, the relationship was particularly strong in patients with increased arterial stiffness or with severe cerebral SVD, supporting the hypothesis that greater transmission of pulsatile pressure to the brain through a stiff aorta is a potentially causative mechanism for SVD. Due to the loss of the Windkessel effect in such patients,^38^ they are more vulnerable to the impact of HR on pulsatility. As such, interventions to increase HR in this patient group provide a potential novel treatment target to reduce PI, and thereby reduce progression of cerebral SVD, with a clinically significant magnitude of this effect suggested by the association between MCA‐PI and SVD severity in this study and between beat‐to‐beat HR and MCA‐PI. This implies a potential 11.7% risk of having a 1 point increase in Fazekas score per 10 bpm HR, in higher risk patients over 70 with a low HR. From previously reported independent associations between MCA‐PI and recurrent cardiovascular events, this would correlate with a potential 13.2% reduction in the risk of cerebrovascular events per 10 bpm.^39^ Effects on cognitive outcomes are uncertain, but could be substantially greater dependent upon the period of exposure.

Increases in resting HR are associated with increased cardiovascular morbidity and mortality in observational studies.^1^ This partly reflects confounding by covariance with other risk factors such as hypertension,^3^, ^4^ but also reflects an increased risk of detrimental cardiac outcomes including heart failure,^40^ myocardial infarction, arrythmias, and sudden cardiac death.^41^ However, these studies largely do not include elderly patients with severe WMH at an increased risk of vascular dementia. Personalisation of treatment through careful selection of patients with potential cerebral benefit from increases in HR and who have limited cardiac risk may identify an important group in whom this presents an opportunity for a new mechanism to reduce SVD and its consequences. This could be achieved by avoidance of rate‐limiting drugs such as beta‐blockers. Although these are now infrequently used for stroke prevention, their cessation when introduced for other indications (migraine, anxiety, coronary ischemia) may be beneficial. Alternatively, addition of drugs that increase HR could be a potential treatment option in patients at low risk of cardiac complications with low HRs. Furthermore, very low diastolic pressures may also have detrimental effects on coronary perfusion, as implied by the possible ‘j‐shaped curve’ for coronary events.^42^

This study does have some limitations. It is an observational study in a selective population of patients with recent TIA or minor stroke, focused on an older age group; therefore, this mechanism cannot be generalized to other populations. However, these are the patients most likely to benefit from interventions to target SVD. Second, not all the patients had TCD performed. This largely reflects the relatively high proportion of elderly patients in the population who are most likely to lack acoustic bone windows. Third, only 87% of the studied population had an MRI scan, as is common in a clinical service due either to patient preference or contraindications. Fourth, the use of multiple antihypertensive medications in combination, and indication bias in which patients are given which drug, prevents reliable conclusions regarding the effects of different antihypertensive classes. However, a greater effect of beta‐blockers on the relationship between HR and MCA‐PI is consistent with the greater relationship between HR and MCA‐PI at lower resting HRs, and with previous evidence for a detrimental effect of beta‐blockers on cerebral pulsatility.^37^ Finally, calculation of beat‐to‐beat relationships is not readily available, limiting its application in clinical practice. However, treatment strategies based upon HR are unlikely to require this complex monitoring as we have demonstrated that the population can be easily stratified simply by age, resting HR, and brain imaging findings.

This is an observational study and therefore does not confirm that there is a causative relationship between HR, cerebral pulsatility, resulting SVD, and subsequent clinical harms, which could only be proven by randomized controlled trials that increase HR. However, by demonstrating a direct, within‐individual, beat‐to‐beat relationship between spontaneous changes in HR or BP with changes in cerebral pulsatility, we can provide stronger support for this postulated mechanism than is possible through cross‐sectional epidemiology. However, interventional studies are required to determine whether there is a causative relationship between low HR, SVD, and clinical outcomes in patients at an increased risk of vascular dementia due to severe SVD; to test whether interventions to alter HR improve cerebral pulstatility in this population; and to pursue randomized trials of heart‐rate increases in patients at an increased risk of vascular dementia.

Our study identifies that in patients with a recent TIA or minor stroke changes in HR are associated with concurrent changes in cerebral pulsatility, with a low HR being a potential key determinant of increased cerebral arterial pulsatility. This association is particularly strong in older patients with lower mean HRs and with severe SVD, likely inducing lower end‐diastolic BP and resulting in low cerebral perfusion between heart beats. Cerebral pulsatility and low end‐diastolic cerebral blood flow are strongly, and potentially causally, associated with cerebral SVD, the most prevalent cause of significant neurological disability, causing both stroke and dementia. As such, bradycardia may explain the less beneficial effects of beta‐blockers on cerebrovascular outcomes, and represents a new potential treatment target for the prevention of stroke and vascular cognitive impairment. Further research is required to test whether this mechanism is evident in non‐stroke patients, whether randomized controlled trials of interventions increasing HR induce changes in cerebral pulsatility, and whether this translates into a reduction in SVD, stroke, and vascular cognitive impairment, without an excessive increase in cardiac morbidity.

## Author Contributions

A.W. and P.M.R. contributed to the conception and design of the study; A.W., K.W., L.L., and K.W. contributed to the acquisition and analysis of data; A.W. and P.M.R. contributed to drafting the text and preparing the figures.

## Potential Conflicts of Interest

There are no conflicts of interest.

## Data Availability

The data that support the findings of this study are available from Prof Rothwell (peter.rothwell@ndcn.ox.ac.uk) upon reasonable request.
